# HIV-1 Mutant Assembly, Processing and Infectivity Expresses Pol Independent of Gag

**DOI:** 10.3390/v12010054

**Published:** 2020-01-02

**Authors:** Fu-Hsien Yu, Kuo-Jung Huang, Chin-Tien Wang

**Affiliations:** 1 Institute of Clinical Medicine, School of Medicine, National Yang-Ming University, Taipei 112, Taiwan; pokpok1011@hotmail.com; 2 Department of Medical Research, Taipei Veterans General Hospital, Taipei 112, Taiwan; kjhuang@vghtpe.gov.tw

**Keywords:** HIV-1, Gag, Pol, Gag–Pol, virus assembly, virus processing

## Abstract

The *pol* retrovirus gene encodes required enzymes for virus replication and maturation. Unlike HIV-1 Pol (expressed as a Gag–Pol fusion protein), foamy virus (described as an ancient retrovirus) expresses Pol without forming Gag–Pol polyproteins. We placed a “self-cleaving” 2A peptide between HIV-1 Gag and Pol. This construct, designated G2AP, is capable of producing virions with the same density as a wild-type (wt) HIV-1 particle. The 2A peptide allows for Pol to be packaged into virions independently from Gag following co-translationally cleaved from Gag. We found that G2AP exhibited only one-third the virus infectivity of the wt, likely due, at least in part, to defects in Pol packaging. Attenuated protease (PR) activity, or a reduction in Pol expression due to the placement of 2A-mediated Pol in a normal Gag–Pol frameshift context, resulted in significant increases in virus yields and/or titers. This suggests that reduced G2AP virus yields were largely due to increased PR activity associated with overexpressed Pol. Our data suggest that HIV-1 adopts a gag/pol ribosomal frameshifting mechanism to support virus assembly via the efficient modulation of Gag–Pol/Gag expression, as well as to promote viral enzyme packaging. Our results help clarify the molecular basis of HIV-1 gene expression and assembly.

## 1. Introduction

The retroviral *pol* gene encodes protease (PR), reverse transcriptase (RT) and integrase (IN); all essential for virus replication [1]. For orthoretroviruses such as HIV-1 and murine leukemia virus (MLV), Pol and the viral structure protein Gag are translated from the same mRNA, with Pol translated as a Gag–Pol fusion protein via ribosomal readthrough or frameshifting. In HIV-1, the 5′ end of the pol reading frame partially overlaps with the 3′ end of the gag reading frame. During Gag precursor Pr55 translation, a -1 ribosomal frameshift occurs at a frequency of approximately 5%, resulting in Pol translation as a Gag–Pol fusion protein at about 5–10% of the Gag expression level [2]. Within Gag–Pol, the C-terminal p6gag domain is truncated and replaced with a transframe region (TFR) known as p6* or p6pol.

During virus assembly, Gag–Pol recruitment for assembling viral particles facilitates Gag–Pol dimerization, which is believed to trigger embedded PR domain activation [3]. Once activated, PR functions as a homodimer and autocleaves from Gag–Pol before mediating the proteolytic processing of Pr55gag and Gag–Pol [4]. Pr55gag cleavage yields four major products: matrix (p17; MA), capsid (CA, p24), nucleocapsid (p7), and C-terminal p6gag [5]. In addition to Gag cleavage products, Gag–Pol processing produces PR, RT and IN.

Gag–Pol/Gag maintenance at a low expression ratio is critical to virus production, since the artificial overexpression of Gag–Pol or Pol containing an active PR markedly reduces virus yields. Reduced yields are likely due to premature or enhanced Gag cleavage via overexpressed PR activity [6,7,8,9,10,11,12]. Gag–Pol interactions with Gag are apparently responsible for Gag–Pol incorporation into virions, with determinants of Gag–Pol viral incorporation largely residing in the N-terminal Gag domain [13,14,15,16]. Unlike orthoretroviruses, foamy virus (FV), considered an ancient retrovirus [17], has no Gag–Pol species [18]; Pol is expressed from separate mRNA [19]. Data from the co-expression of Gag–Pol and Gag from separate plasmids indicate that both HIV-1 and MLV Gag–Pol lacking the Gag domain are still capable of incorporation into Gag virus-like particles (VLPs) [20,21,22]. These findings suggest the possibility of interactions involving Pol and Gag for HIV, MLV and FV, with an undefined Pol incorporation mechanism shared by all three retroviruses. However, FV Pol apparently does not significantly affect virus assembly, despite independent Gag expression. In contrast, there is potential for a significant reduction in HIV-1 virus yields if Gag–Pol or Pol is expressed at levels above normal physiological Gag–Pol/Gag ratios [21].

It is likely that the regulation of FV PR-mediated virus processing differs from that found in HIV. Alternatively (and not mutually exclusive), HIV-1 PR may possess stronger enzymatic activity than its FV counterpart in terms of mediating virus particle processing. One possibility is that an HIV-1 ribosomal frameshift system has evolved to express Pol as a Gag–Pol fusion protein with selectively stronger enzyme activity, despite a marked reduction in expression level. Accordingly, HIV-1 Gag–Pol, even when expressed at a relatively low level, may be capable of efficient packaging, thus providing sufficient enzyme activity for virus replication. The conclusion that HIV-1 Pol can be incorporated into Gag VLPs is largely based on a co-expression system involving the co-transfection of PR-defective Pol and Gag expression vectors [20,21]. Since PR-mediated virus maturation is essential for virus infectivity, the biological relevance of PR-defective Pol is unclear. A major limitation of a co-expression system involving Pr55gag and HIV-1 PR-active Pol is that Pr55gag particles can bud from cells lacking PR-active Pol co-expression, making it difficult to determine virus processing efficiency. For the present study we engineered a construct capable of expressing HIV-1 Gag and Pol from the same mRNA. Although this construct is capable of producing infectious virus particles, its virus titer, virus-associated RT, and genomic RNA levels are significantly lower compared to the wild type. Our results support the idea that HIV-1 exploits a gag/pol ribosomal frameshifting system to promote virus assembly and replication.

## 2. Materials and Methods

### 2.1. Plasmid Construction

**Figure 1:**
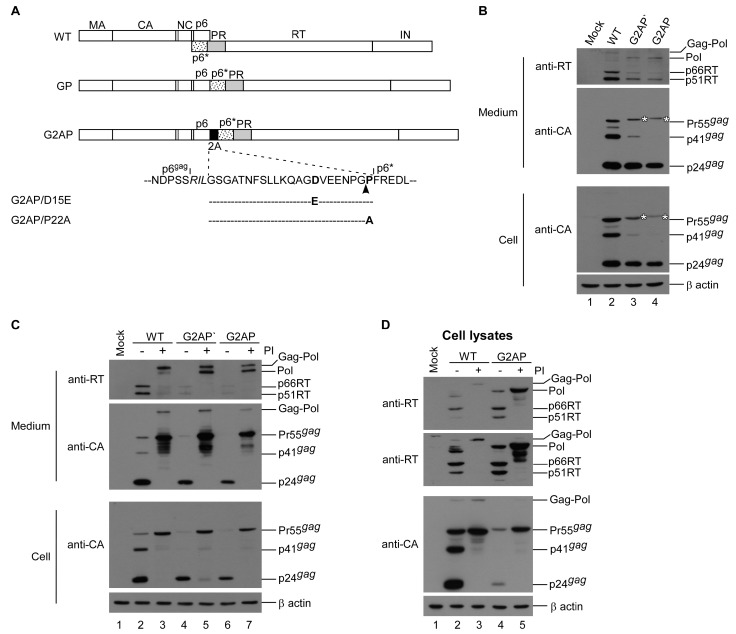
Assembly and processing of HIV-1 Gag and Pol expressed from a single plasmid. (**A**) Schematic representations of HIV-1 Gag and Gag–Pol expression constructs. Indicated are the HIV-1 Gag protein domains MA (matrix), CA (capsid), NC (nucleocapsid), p6, 2A peptide sequence and *pol*-encoded p6*, PR, RT and IN. Arrowhead indicates 2A cleavage site. Altered or additional residues in italics. G2AP/D15E and G2AP/P22A are 2A substitution mutants, with a Glu substitution for Asp at position 15 and an Ala substitution for Pro at position 22, respectively. (**B**) G2AP assembly and processing. HEK 293T cells were transfected with designated construct. G2AP’ is identical to G2AP except for a Phe substitution for Ser-465 in Gag. Cells and supernatants were collected 48 h post-transfection and analyzed by Western immunoblotting. Membrane-bound proteins were initially probed with anti-RT serum prior to stripping and probing with an anti-p24CA monoclonal antibody. HIV-1 Gag–Pol, Pol, 66/51RT, Pr55gag, p41gag and p24gag positions are shown. Asterisks denote Gag-2A positions. (**C**) HIV-1 protease activity suppression increased G2AP virus production. HEK 293T cells were transfected with designated constructs. At 4 h post-transfection, equal amounts of cells were plated on two dishes and either left untreated or treated with saquinavir (an HIV-1 protease inhibitor) at a concentration of 5 μM. Supernatants and cells were collected 48 h post-transfection, prepared, and subjected to Western immunoblotting. (**D**) G2AP expressed Pol and Pr55gag at comparable levels. Cell lysates derived from panel C were probed with HIV-1 RT antiserum. For cellular background reduction, anti-RT serum was pre-incubated with membranes containing mock-transfected cell lysates. Middle panel: Images from longer exposures of the upper panel blot. Gag–Pol, Pol, 66/51RT and Gag protein positions are shown.

G2AP was constructed by placing a 2A peptide coding sequence between the Gag C-terminus and Pol N-terminus (Figure 1), using 5′-CCGGATCCTAGGAAGCGGAGCTACTAACTTCAGCCTGCTTAAGCAGGCTGGAGACGTGGAGGAGAACCCTGGACCTTTTAGGGAAGATCTGGCC-3′ as the forward primer and 5′-GGTACAGTCTCAATAGGGCTAATG-3′ as the reverse primer (HIV-1 nt.2575-52). The forward primer contains a flanking BamHI site, a 2A-like peptide (underlined), and partial N-terminal p6* coding sequences. HIVgpt served as a template. The 2A peptide coding sequence is from porcine teschovirus-1 (*Picornaviridae* virus family), described as having the highest cleaving efficiency among picornavirus 2A species tested in cell cultures [23]. Amplified fragments were digested with *BamHI* and *BclI* prior to cloning into GP [24]. GP (previously designated Gagp6-Pol) contains a p6gag-intact full-length Gag–Pol with a TCCCGCG substitution for the slippery sequence TTTTTTA, thereby preventing Gag–Pol frameshifting [25]. Two BglII-containing reverse primers were used to amplify 2A-coding fragments containing the designated mutation: P22A (5′-CCAGA TCTTC CCTAA AGGCG CCAGG GTTC-3′) and D15E (5′-CCAGA TCTTC CCTAA AGGGC CCAGG GTTCT CCTCC ACCTC TCCAG CCTG-3′). G2AP served as a template. The forward primer sequence was 5′-GGAAC TACTA GTACC CTTCA GGAAC AA-3′ (nt. 1500-27). PCR-amplified fragments were purified, digested with BglII, and cloned into a G2AP mutation containing a pBRClaSal plasmid cassette spanning the HIV-1 proviral sequence from ClaI-nt. 831 to SalI-nt. 5786. Each resultant plasmid was digested with ClaI and SalI and cloned into HIVgpt, yielding G2AP/P22A and G2AP/D15E. Next, 2A-, P22A- and D15E-coding fragments were PCR-amplified with a 5′-CGTCA CGGAT CCTGG AAGCG GAGC-3′ forward primer and 5′-TACAT ACAAA TCATC CATGT ATTGA TA-3′ reverse primer (HIV-1 nt. 3116-90). G2AP, G2AP/P22A and G2AP/D15E served as templates. Amplified coding fragments were purified, digested with BamHI and EcoRV, and subcloned into a pBRClaSal/DPR cassette [26]. Resultant constructs were digested with SpeI and SalI and ligated into HIVgpt, yielding D2APol, D2A/P22APol, and D2A/D15EPol, respectively. PR/F99V (containing a Val substitution for the final C-terminal PR Phe residue) was constructed with an overlap extension PCR using a 5′-GGTACAGTCTCAATAGGGCTAATG-3′ primer containing the desired mutation (F99V). PR/F99V was digested with BclI and SalI and cloned into G2AP, yielding G2AP/F99V. G2AP/T26S was derived from a recombination of G2AP and the PR activity-diminished mutation T26S [10]. GPfs, as described previously, a Pr160Gag–Pol expression plasmid contains a frameshift mutation at the gag/pol junction [21].

### 2.2. Cell Culture, Transfection, and Infection

HEK 293T and HeLa cells [27] were maintained in Dulbecco’s Modified Eagle Medium (DMEM) containing 10% fetal calf serum. Confluent HEK 293T cells were trypsinized and split 1:10 before being added to 10 cm dish plates 16–24 h pre-transfection. For HEK 293T cell transfection, calcium phosphate precipitation was employed using 20 µg of each construct’s DNA plasmids. Transfection efficiency was increased via co-culturing with 50 μM chloroquine.

Virus infectivity was determined by single-round infection assays involving the co-transfection of 5 μg of the VSV-G protein expression vector pHCMV-G [28] with either 10 µg of a wt or mutant HIVgpt, or 10 µg Gag plus 1 µg of a Gag–Pol expression vector. After collecting and filtering virus-containing supernatants, filtrate aliquots were diluted between 1:10 and 1:100 and used to infect HeLa cells. Polybrene (4 μg/mL) was added to enhance both virus adsorption and transduction efficiency. Transfectants and remaining supernatants were prepared and subjected to Western immunoblotting. At 24 h post-infection, cells were trypsinized and split prior to placement onto dishes. Infected cells were treated with medium containing a drug selection cocktail [27]. Colonies formed by drug-resistant cells were fixed and stained with 50% methanol containing 0.5% methylene blue prior to titer conversion (cfu/mL). Mutant infectivity percentages were calculated by dividing total mutant titers by wt titers in parallel experiments and multiplying by 100.

### 2.3. Sucrose Density Gradient Fractionation

Transfected HEK 293T cell supernatant was collected, filtered, and centrifuged through a 2 mL 20% (*w*/*v*) sucrose cushion as described above. Viral pellets were suspended in TSE buffer (10 mM Tris-HCl at pH 7.5, 100 mM NaCl, 1 mM EDTA) and overlaid on a 20–60% (*w*/*v*) sucrose gradient consisting of 1 mL layers of 20, 30, 40, 50 and 60% (*w*/*v*) sucrose in TSE buffer that had sat for 2 h. Gradients were centrifuged in an SW50.1 rotor at 40,000 rpm (274,000× *g*) for 16 h at 4 °C; 500 μL fractions were collected from top to bottom. Sucrose density was measured for each fraction. Fraction proteins were precipitated with 10% trichloroacetic acid (TCA) and subjected to Western immunoblotting.

### 2.4. Western Immunoblot Analysis

Cells and supernatants from transfected HEK 293T cells were prepared for Western immunoblotting as described previously [26]. Briefly, supernatants were filtered and centrifuged through 2 mL of 20% (*w*/*v*) sucrose at 274,000× *g*. Cells were rinsed with ice-cold phosphate-buffered saline (PBS), collected in 1 mL PBS, and pelleted. Viral and cell pellets were suspended in IPB buffer (20 mM Tris-HCl at pH 7.5, 150 mM NaCl, 1 mM EDTA, 0.1% SDS, 0.5% sodium deoxycholate, 1% Triton X-100, 0.02% sodium azide) containing 0.1 mM phenylmethylsulfonylfluoride (PMSF). Supernatant and cell samples were mixed with equal volumes of 2× sample buffer (12.5 mM Tris-HCl at pH 6.8, 2% SDS, 20% glycerol, 0.25% bromophenol blue) containing β-mercaptoethanol (5%) and boiled for 5 min. Samples were subjected to 10% SDS-PAGE and electroblotted on nitrocellulose membranes. Membrane-bound Gag and RT detection protocols were performed as previously described [26]. Membrane-bound HIV-1 Gag/Gag–Pol or 2A-tagged Gag were probed using an anti-p24gag (mouse hybridoma clone183-H12-5C) or anti-2A monoclonal antibody (Novus Biologicals, Centennial, CO, USA). Rabbit antiserum or mouse anti-RT monoclonal antibodies served as primary antibodies for HIV-1 RT or RT-associated Pol [29,30]. Secondary antibodies were either sheep anti-mouse or goat anti-rabbit (HRP)-conjugated (Jackson ImmnunoResearch, West Grove, PA, USA). Membrane-bound proteins were found using an enhanced chemiluminescence (ECL) detection system according to the manufacturer’s protocols (Thermo Fisher Scientific Waltham, MA, USA).

## 3. Results

### 3.1. HIV-1 Gag and Pol Expression in a Single Plasmid

To directly observe the impacts of PR-active Pol on virus assembly, processing, and infectivity, a construct designated G2AP was engineered by placing a “self-cleaving” 2A peptide coding sequence between the Gag C-terminus and Pol N-terminus (Figure 1A). A cotranslational cleavage event (more precisely described as ribosomal skipping) is believed to occur at the 2A C-terminus [31], resulting in Gag-independent Pol translation. To prevent ribosomal frameshifting at the gag/pol junction, the original slippery sequence TTT TTA within G2AP and G2AP-derived constructs was changed to CCC GCG, thus blocking a -1 ribosomal frameshift event [25]. Results from transient expression in HEK 293T cells indicate that G2AP and G2AP’ (a G2AP version containing a change in Ser465Phe in Gag due to PCR-mediated cloning) are capable of assembling and processing virus particles, with virus-associated RT and Pol readily detectable (Figure 1B upper panel, lanes 3 and 4). The bands that migrated slightly slower than Pr55gag correspond to the molecular weight of 2A-tagged Pr55gag (Figure 1B, asterisks). Both G2AP and G2AP’ consistently exhibited lower virus-associated Gag and RT levels compared to the wt, likely due to enhanced Gag cleavage by overexpressed Pol.

If 2A is capable of efficiently mediating cleavage involving Gag and Pol, Pol might represent a major virus-associated RT precursor when PR activity is blocked. To test this possibility, we added an HIV-1 protease inhibitor to culture medium following transfection. As shown in Figure 1C, RT-associated Pol was readily detected in virions when treated with an HIV-1 PR inhibitor (lanes 5 and 7, upper panel). However, bands corresponding to Gag–Pol were also observed in G2AP and G2AP’ supernatant samples following treatment with a protease inhibitor, suggesting that ribosomes may read through 2A, but at very low frequencies. However, cellular G2AP Gag–Pol was still barely detectable at the end of a longer exposure (Figure 1D middle panel). Since wt Gag–Pol was readily detectable following treatment with an HIV-1 PR inhibitor, our observation that cellular G2AP Gag–Pol was barely detected under the same conditions (Figure 1D middle panel, lane 3 vs. lane 5) suggests a very low frequency for a 2A ribosomal readthrough. If any translated G2AP Gag–Pol is present, it might be packaged into viral particles at a higher frequency than Pol, resulting in a significant decrease in the detection of cellular G2AP Gag–Pol. This would be consistent with a scenario in which Gag–Pol is packaged more efficiently than Pol.

### 3.2. G2AP Exhibits Wild-Type HIV-1 Particle Density and Possesses Infectivity

**Figure 2:**
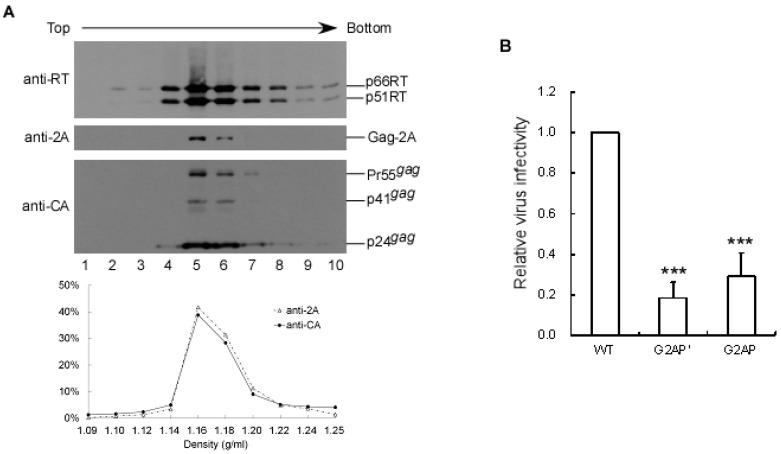
G2AP exhibits wild-type HIV-1 particle density and possesses infectivity. (**A**) Sucrose density gradient fractionation of G2AP particles. HEK 293T cells were transfected with a wt or G2AP expression vector. Supernatants were collected and pelleted through 20% sucrose cushions 48–72 h post-transfection. Viral pellets were resuspended in PBS. To make direct comparisons with wt HIV-1 particle density, G2AP pellets were spun with wt pellets through the same sucrose density gradient (20–60%) for 16 h. Ten fractions (equal quantities) were collected from top to bottom. Fraction densities were measured, and virus proteins analyzed by Western immunoblotting sequentially probed with 2A, p24gag, and RT antibodies. 2A- and p24gag-associated Gag proteins in each fraction were quantified by scanning immunoblot band densities. Relative 2A- and p24gag-associated protein levels in each fraction were plotted against sucrose densities. (**B**) Infectivity of HIV-1 mutants. HEK 293T cells were cotransfected with a designated construct plus a VSV-G expression vector. Supernatants were collected, filtered, and used to infect HeLa cells 48 h post-transfection. Drug-resistant colony infection and selection were performed as described in Materials and Methods section. Mutant infectivity was determined as the ratio of mutant titers to wt titers, normalized to Gag protein levels in parallel experiments. *** *p* < 0.001.

Since collected medium was filtered and centrifuged through 20% sucrose cushions, we posited that Gag and RT in culture medium was virus-associated. To confirm the presence of released G2AP Gag as virus particles, pellets derived from G2AP transfectant supernatant were centrifuged with wt viral pellets through the same sucrose density gradient. As shown in Figure 2A, both G2AP and wt Gag peaked at the same fraction with a sucrose density of 1.16 g/mL, which is consistent with a wt HIV-1 particle density. When pseudotyped with VSV-G, G2AP and G2AP’ displayed virus infectivity at 20–30% of wt levels (Figure 2B). This suggests G2AP Pol packaging into virions and the consequent provision of enzymes required for virus replication.

**Figure 3:**
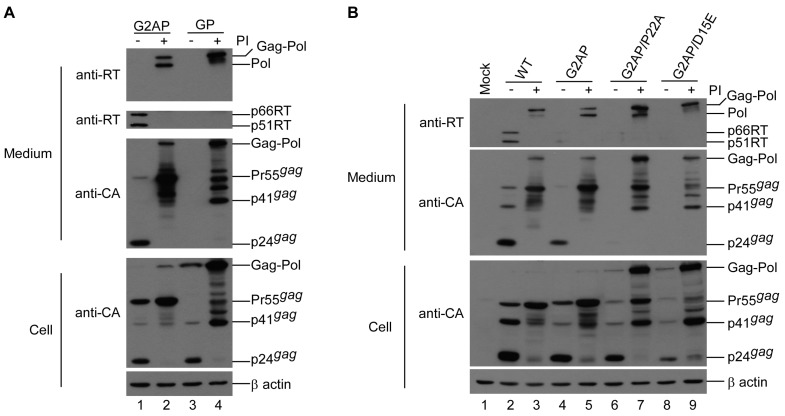
Expression of Pol from G2AP is 2A-dependent. (**A**,**B**) HEK 293T cells were transfected with the designated construct. Except for the absence of the 2A peptide, GP is identical to G2AP. At 4 h post-transfection, equal amounts of cells were plated on two dishes and either left untreated or treated with saquinavir, an HIV-1 protease inhibitor. Supernatants and cells were collected 48 h post-transfection, prepared, and subjected to Western immunoblotting as described in the Figure 1 caption.

A 2A-deficient counterpart designated GP was constructed to confirm that the 2A cleavage function gives G2AP the ability to express Pol independently of the gag reading frame. In addition, 2A mutants defective in cleavage activity [32] were cloned into G2AP, resulting in constructs designated G2AP/D15E and G2AP/P22A (Figure 1A). In contrast to G2AP, GP had barely detectable RT and Gag proteins in supernatant, but readily detectable Gag–Pol in cellular samples (Figure 3A, lane 1 vs. lane 3). Treating GP transfectants with saquinavir resulted in substantial amounts of Gag products associated with Gag–Pol and p24 in supernatant samples (Figure 3A, lane 4). This is consistent with an earlier finding that the failure of GP to assemble and release from cells is due to enhanced autocleavage function, since GP VLPs become readily detected in medium when PR is mutationally inactivated [24]. This is likely due to the incomplete suppression of overexpressed PR activity; unprocessed or partially processed Gag might assemble and release from GP transfectants (Figure 3A, lane 4). Similar to GP, G2AP/D15E and G2AP/P22A exhibited barely detectable Gag and RT-associated products in supernatants. However, following treatment with saquinavir, they produced substantial amounts of RT-associated Pol, Gag–Pol, and Gag products in both medium and cell samples (Figure 3B, lanes 6–9). In addition to Gag–Pol, Pol was readily detected in G2AP/P22A transfectant supernatant samples, but barely detected in G2AP/D15E (Figure 3B upper panel lane 7 vs. lane 9), suggesting that the D15E mutation is more effective than the P22A mutation in terms of 2A cleavage activity inhibition.

### 3.3. Reduced Pol Expression Significantly Increases Virus Yields

**Figure 4:**
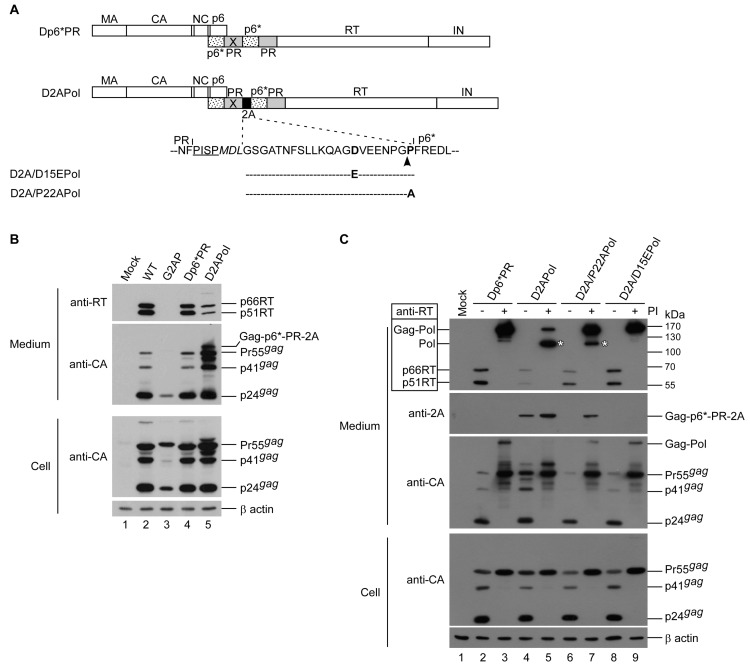
Effects of reduced Pol expression on virus assembly and processing. (**A**) Schematic representations of HIV-1 Gag and Gag–Pol expression constructs. HIV-1 Gag protein domains and pol-encoded proteins are indicated as described in the Figure 1 caption. X denotes a PR-inactivated mutation. Amino acid residues are denoted at 2A-flanking PR cleavage sites. Underlined PISP indicates remaining N-terminal RT residues. Altered or additional residues are in italics. (**B**,**C**) Insufficient viral incorporation of PR-active Pol. HEK 293T cells were transfected with designated constructs. Panel B: At 4 h post-transfection, equal amounts of cells were plated on two dishes and either left untreated or treated with saquinavir, an HIV-1 protease inhibitor. Supernatants and cells were collected 48 h post-transfection, prepared, and subjected to Western immunoblotting. Asterisks indicate Pol positions. Asterisks denote the Pol positions.

The G2AP production of virus-associated Gag and RT at levels much lower than those of the wt was in part due to enhanced Gag cleavage by overexpressed Pol containing active PR. Accordingly, virus yields can increase if the Pol expression level decreases to a normal Gag–Pol/Gag ratio. To confirm this assumption, we used an HIV-1 virus-producing vector (designated Dp6*PR) previously described as capable of virus particle assembly and processing in a manner similar to that of a wt [27]. Dp6*PR expressed Gag–Pol containing duplicated p6*-PR, with the first PR copy intentionally mutated to remove enzymatic activity. An in-frame insertion of 2A between the PR-inactivated PR and the pol coding sequence within Dp6*PR yielded a construct labeled D2APol (Figure 4A). Since the original gag/pol frameshift signal remained intact, the level of 2A-mediated Pol expression from D2APol was significantly reduced to 5–10% of Gag. D2APol produced virus-associated Gag at a level much higher than that of G2AP (Figure 4B middle panel, lane 5 vs. lane 3), supporting the assumption that G2AP virus yields could be increased when the Pol expression level decreases to a normal Gag–Pol/Gag ratio. D15E and P22A were cloned into D2APol (designated D2A/D15EPol and D2A/P22APol) to serve as controls. According to our Western blot results, Dp6*PR exhibited a phenotype similar to that of a wt (Figure 4B, lane 2 vs. lane 4), which is consistent with previous reports [26,27]. In contrast, D2APol had higher Gag but lower RT levels in supernatant samples compared to Dp6*PR. This suggests a defect in Pol incorporation into virions (Figure 4B, lane 5). Deficient virus-associated Pol might fail to provide sufficient PR activity to mediate virus processing, which would explain why a considerable amount of D2APol virus-associated Gag remained unprocessed or incompletely processed. Deficient RT and insufficient virus maturation led to poor D2APol infectivity (Table 1). Similar to G2AP, a trace of Gag–Pol was detected in a D2APol transfectant supernatant sample in the presence of an HIV-1 PR inhibitor (Figure 4C upper panel, lane 5). Since D15E is capable of blocking 2A function (Figure 3B), we were not surprised to observe that D2A/D15EPol exhibited a phenotype similar to that of Dp6*PR (Figure 4B, lanes 2–3 and 8–9). Similar to G2AP/P22A, Pol was still detectable in D2A/P22APol transfectant supernatant samples when PR activity was suppressed (Figure 4C, upper panel lane 7), due to incomplete inhibition of 2A function by the P22A mutation.

A band migrating slower than Pr55gag might consist of Gag(MA-CA-NC)-p6*-PR-2A (Figure 4B,C upper second panels) derived from 2A-mediated cleavage during D2APol Gag–Pol translation. There is a possibility that this 2A-associated Gag product assembles and releases from cells as VLPs. Combined, these results (a) support a hypothesis that reduced Pol expression level ameliorates the virus assembly defect incurred by Pol overexpression, and (b) support the idea that Pol expression as a Gag–Pol fusion protein is required for efficient viral enzyme incorporation into virus particles.

### 3.4. PR Activity Attenuation Increases Virus Titers

**Figure 5:**
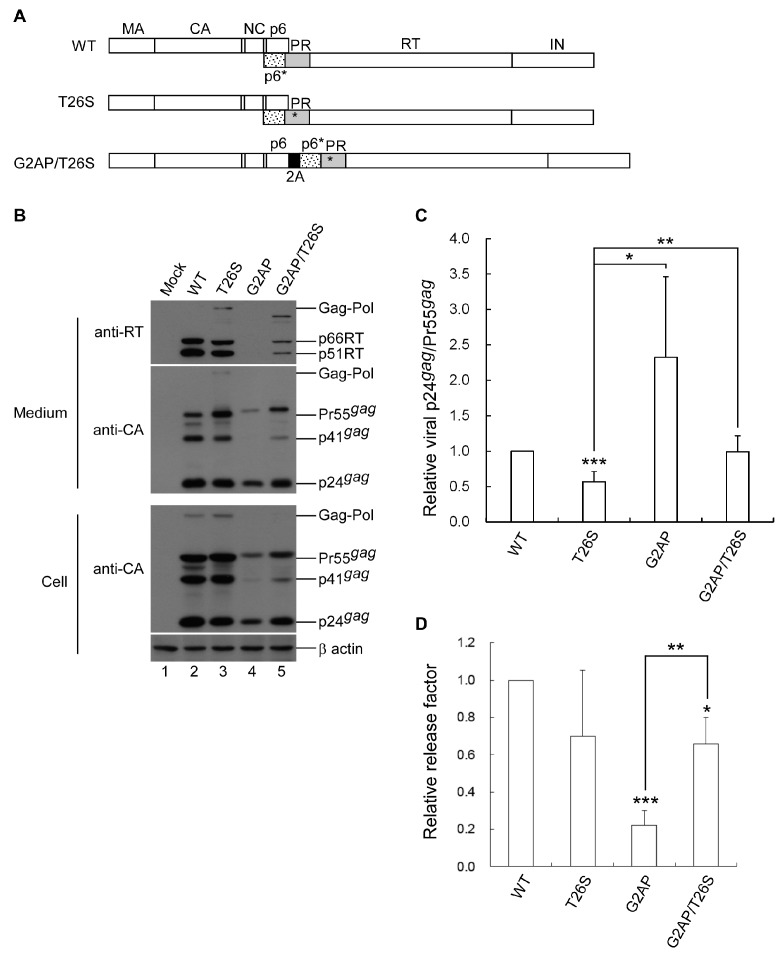
PR activity attenuation enhances G2AP virus titers. (**A**) Schematic representations of HIV-1 Gag and Gag–Pol expression constructs. Indicated are HIV-1 Gag protein domains, pol-encoded proteins, and the 2A peptide as described in the Figure 1 caption. Asterisks denote the T26S mutation, in which Ser is substituted for the Thr26 of PR. (**B**) HEK 293T cells were transfected with a designated construct. Two days post-transfection, supernatants and cells were collected, prepared, and subjected to Western blot analysis. (**C**) Virus particle processing efficiency data. Quantities of Pr55gag and mature p24gag were measured by scanning their respective band densities from immunoblots. p24gag-to-Pr55gag quantity ratios were calculated for each construct and compared with wt ratios—that is, the p24gag/Pr55gag ratio for each mutant was divided by the wt p24gag/Pr55gag ratio in parallel experiments. Error bars indicate standard deviations. * *p* < 0.05; ** *p* < 0.01; *** *p* < 0.001. (**D**) Relative virus release efficiency. Virus-associated or cellular Gag protein levels were quantified by scanning p24gag-associated band densities from immunoblots. Ratios of total virus-associated Gag protein level to total cellular Gag protein level were calculated and compared to the wt by dividing the ratio for each mutant by the ratio for the wt in parallel experiments. Error bars indicate standard deviation. * *p* < 0.05; ** *p* < 0.01; *** *p* < 0.001.

Our results strongly support the proposal that overexpressed PR activity due to increased Pol expression impedes virus assembly [9,10,11]. We therefore posited that attenuation of the PR activity of overexpressed Pol may reduce the G2AP virus assembly defect. To test this idea, we engineered a PR mutant T26S (with a Ser replacement for Thr at position 26 in the PR domain) and cloned T26S into G2AP, yielding G2AP/T26S (Figure 5A). The T26S mutation has been reported as triggering a 4-fold reduction in PR activity [10]. According to our transient expression data, T26S exhibited a significant decrease in the virus-associated p24gag/Pr55gag ratio with detectable unprocessed Gag–Pol (Figure 5B, lane 3 vs. lane 2 and Figure 5C), which is consistent with a PR activity deficiency imposed by a T26S mutation. Compared to G2AP, G2AP/T26S exhibited readily detectable virus-associated RT (Figure 5B) and a significant increase in virus yield (Figure 5D).

The detection of virus-associated Pol in G2AP/T26S also reflects impaired PR activity due to the T26S mutation (Figure 5B upper panel, lane 5). Results from single-cycle infection assays indicate significant increases in G2AP virus titers, which is in agreement with increased virus yields following T26S mutation (Table 2).

### 3.5. PR-RT Cleavage Blocking Enhances *G2AP* Virus Yields

**Figure 6:**
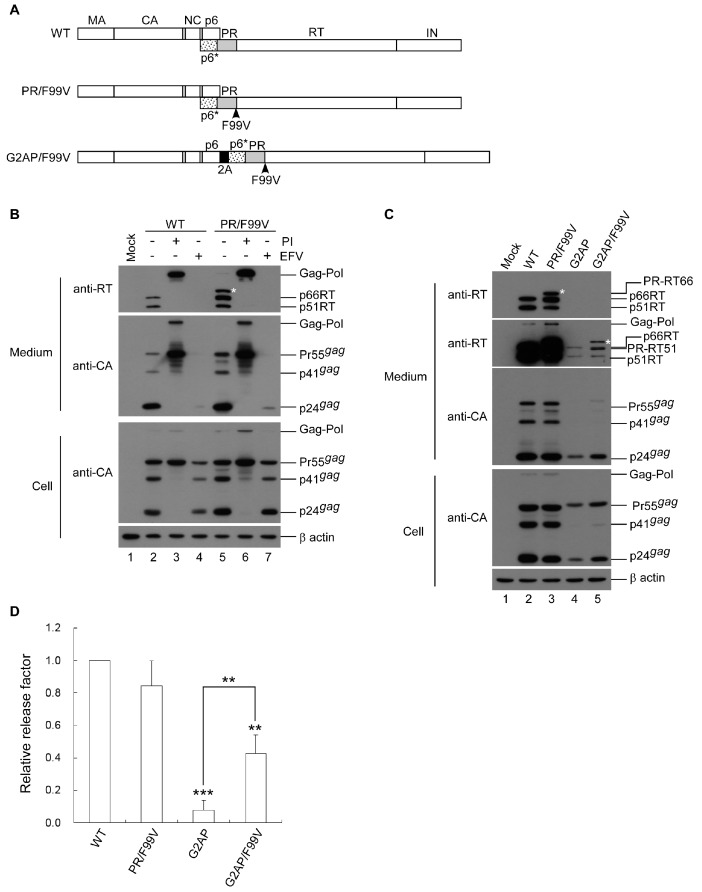
Blocking PR-RT cleavage significantly enhances G2AP virus titers. (**A**) Schematic representations of HIV-1 Gag and Gag–Pol expression constructs. HIV-1 Gag protein domains and pol-encoded proteins are indicated as described in the Figure 1 caption. F99V denotes blocking mutation at the PR/RT site, with the Phe residue at the PR C-terminus replaced with Val. (**B**,**C**) HEK 293T cells were transfected with designated constructs. Panel B: At 4 h post-transfection, equal amounts of cells were plated on three dishes and either left untreated, treated with the HIV-1 protease inhibitor saquinavir (lanes 3 and 6), or with the RT-dimerization enhancer efavirenz (lanes 4 and 7). Cells and supernatants were collected 48 h post-transfection and analyzed by Western immunoblotting. Membrane-bound proteins were probed with anti-p24CA and anti-RT antibodies. Asterisks indicate positions of PR linked with p66RT. Virus assembly efficiency (**D**) for each mutant was determined as described in the Figure 5 caption. Error bars indicate standard deviation. ** *p* < 0.01; *** *p* < 0.001.

An earlier study reported that a mutation preventing PR-RT cleavage did not significantly affect HIV-1 Gag–Pol viral incorporation or virus particle processing [33]. FV Pol contains a single cleavage site between RT and IN [34], suggesting that PR-RT cleavage is not required for FV replication. To clarify the impact of HIV-1 Pol containing a PR-RT fusion on virus assembly and processing, we created a G2AP-derived construct (designated G2AP/F99V) containing a mutation-blocking cleavage at PR-RT (Figure 6A). The PR-RT cleavage mutation was also cloned into a wt HIVgpt; the resultant construct (designated PR/F99V, serving as a control) exhibited a wt Gag assembly and processing profile. Bands migrating to a position corresponding to the PR-RT fusion molecular weight were readily detected in PR/F99V transfectant supernatant samples, indicating that the mutation blocked cleaving at the PR-RT junction (Figure 6B, lane 5). We observed that both wt and PR/F99V protease activity were sensitive to inhibition by the same HIV-1 PR inhibitor. Further, both were susceptible to an efavirenz (EFV) effect that resulted in reduced HIV-1 virus production (Figure 6B). EFV, a non-nucleoside reverse transcriptase inhibitor (NNRTI), is known to facilitate HIV-1 Gag–Pol dimerization, leading to markedly reduced virus production as a result of Gag cleavage enhancement [35,36].

Virus-associated Gag or RT produced by G2AP transfectants increased noticeably when PR-RT cleavage was blocked (Figure 6C, lane 4 vs. lane 5, and Figure 6D). Bands corresponding to PR-RT66 and PR-RT51 fusion molecules were detected in G2AP/F99V transfectant supernatant samples when exposure times were extended (Figure 6C, top second panel, lane 5). Increases in G2AP virus yields due to PR-RT cleavage blocking were linked to increases in virus titers (Table 3). G2AP/F99V expressed significantly higher virus titers than G2AP, which fits with the observation that G2AP/F99V produced virus-associated Gag at a higher level compared to G2AP (Figure 6D). The higher level of virus-associated Gag in G2AP/F99V is likely due, at least in part, to lower Gag cleavage enhancement. Given that the Gag domain contributes to PR activation by facilitating Gag–Pol dimerization, it is likely that the subtle deleterious effect of PR-RT fusion on PR activation may become noticeable in the absence of an upstream Gag domain, as observed in the case of G2AP/F99V.

### 3.6. G2AP Is Defective in Pol Incorporation

**Figure 7:**
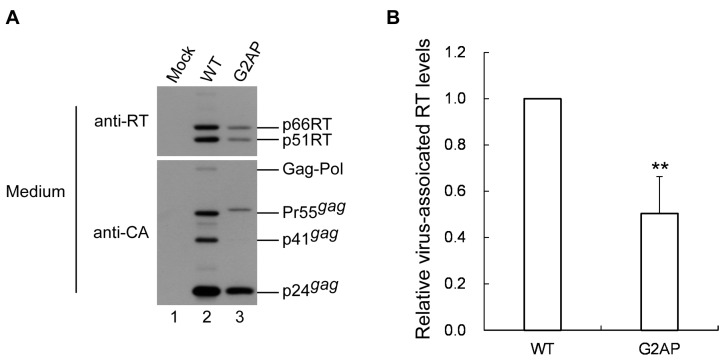
Analysis of G2AP particle RT content. (**A**) HEK 293T cells were transfected with wt or G2AP expression vectors. At 2–3 days post-transfection, supernatants were collected, filtered, and centrifuged through 20% sucrose cushions. Viral pellets from equal amounts of supernatants were resuspended, prepared, and subjected to Western immunoblotting. HIV-1 Gag and Pol proteins were respectively probed with anti-p24CA and anti-HIV-1 RT polyclonal antibodies. (**B**) Virus-associated RT levels. Quantities of p24gag-associated Gag (Pr55gag, p41gag and p24gag) and RT-associated Pol, Gag–Pol and p66/51RT in each sample were quantified using immunoblot scanning band densities. Ratios of total Pol to Gag protein levels were calculated for G2AP and normalized to those of a wt in parallel experiments. Data are from three independent experiments. Error bars indicate standard deviation. ** *p* < 0.01.

Although G2AP exhibited a wt-like particle processing pattern, data from a single-cycle infection assay show that fewer than one-third of the released G2AP virions were infectious (Figure 2B). Deficiencies in virus-associated RT can contribute to reduced virus infectivity. To examine this possibility, aliquots of G2AP and wt viral pellets were subject to Western immunoblotting (Figure 7A) and virus-associated RT quantification. Results indicate that RT-associated proteins in G2AP particles were approximately one-half those in the wt (Figure 7B).

### 3.7. 2A exerts no Major Effects on Post-Assembly Post-Processing Stages of Virus Infectivity

**Figure 8:**
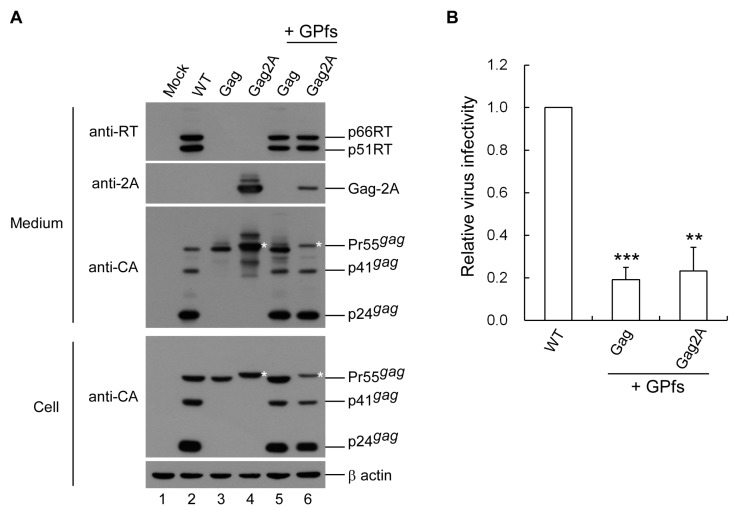
2A does not significantly affect post-assembly post-processing stage of virus replication. HEK 293T cells were transfected with Pr55gag or a 2A-tagged Pr55gag expression vector alone or with a Gag–Pol (GPfs) expression vector at a plasmid DNA ratio of 10:1. Additional VSVG-expressing plasmids were cotransfected for infection assays. At 48–72 h post-transfection, HeLa cells were infected with aliquots of supernatants and subjected to Western immunoblotting (sequentially probed with anti-2A, anti-RT and anti-p24CA monoclonal antibodies) (**A**). Asterisks denote Gag-2A positions. (**B**) Virus infectivity was determined as described in the Figure 2 caption. Error bars indicate standard deviation. ** *p* < 0.01; *** *p* < 0.001.

In addition to playing a role in virus assembly and budding, Gag is also functionally involved in the post-assembly and post-processing steps of virus replication [37]. The capability of G2AP to assemble and process virus particles suggests that a 2A tag at the Gag C-terminus does not affect HIV-1 Gag assembly and processing. However, it is possible that Gag functions involved in the post-assembly and post-processing stages are disturbed by 2A, and therefore contribute to reduced G2AP infectivity. To test this possibility, we constructed a Gag-2A expression vector and coexpressed Gag2A with the Gag–Pol expression vector GPfs. Results indicate that similar to Pr55gag, Gag-2A was processed and contained RT at a comparable level when Gag2A and Pr55gag were coexpressed with GPfs (Figure 8A). Results from three independent experiments show no statistically significant differences in virus titers and infectivity (normalized to virus-associated Gag) between Gag2A and Pr55gag when they were co-expressed with GPfs plus VSV-G (Figure 8B), suggesting that 2A tagged at the Pr55gag C-terminus does not significantly impact the post-assembly post-processing stages of virus replication.

## 4. Discussion

Multiple 2A peptide-like coding sequences are capable of mediating expressions of two or more proteins from a single mRNA template when placed between specific protein coding sequences [23]. To achieve HIV-1 Gag and Pol expression from a single plasmid at equivalent levels, we inserted a 2A peptide coding sequence between Gag and Pol reading frames. Our finding that G2AP-associated Gag–Pol was detectable when PR activity was inhibited suggests incomplete 2A-mediated Pol expression independent of Gag. Since the N-terminal flanking sequence may affect 2A-mediated cleaving [38,39], it is likely that the 2A within G2AP is not fully functional, resulting in the generation of Gag–Pol via a translational readthrough. Still, G2AP apparently expressed roughly equivalent amounts of cellular Gag and Pol (Figure 1D). Further, a substitute mutation of the 2A conserved residue (D15E) significantly reduced the 2A cleavage function (Figure 2). Combined, the results indicate that 2A placement between Gag and Pol contributes to Pol expression independent of Gag.

Our finding of a significant reduction in G2AP virus yield compared to a wt is in agreement with the idea that strict maintenance of the Gag–Pol/Gag expression ratio at low levels is essential for virus assembly. Our finding that both G2AP and D2APol virus-associated RT levels were lower than that of the wt is consistent with the idea that efficient Gag–Pol incorporation into virus particles is largely dependent on interactions between Gag and the Gag–Pol N-terminal Gag domain. Note that the Gag–Pol fusion protein from D2APol was cleaved by 2A into MA-CA-NC-p6*-(defective) PR-2A and Pol. We previously reported that MA-CA-NC-p6*-PR is capable of incorporation into viral particles as well as Gag processing mediation, although the latter is less efficient compared to mediation by PR derived from Gag–Pol [40]. MA-CA-NC-p6*-PR is also capable of assembly and release from cells as virus-like particles (VLPs) when PR is inactivated [24]. Accordingly, it may be that MA-CA-NC-p6*-(defective) PR-2A competes with Pol for viral incorporation, with the defective PR domain interfering with PR function when PR is embedded in Pol. These scenarios would explain, in part, the insufficient D2APol virus particle processing we observed.

Deficient RT is one possible explanation for the reduced G2AP virus infectivity we observed (Figure 7). In addition to the canonical packaging signal located at the 5′ untranslated region and extending to the 5′ terminus of the gag coding sequence [41,42,43,44,45,46,47], a secondary RNA structure involving a ribosomal frameshift signal is required for efficient HIV-1 genomic RNA packaging [48]. Chamanian et al. found that mutations at a HIV-1 gag/pol ribosomal frameshift signal significantly reduced viral genomic RNA packaging efficiency [48]. Since G2AP contains a substitution mutation at the ribosomal frameshift slippery sequence TTTTTT, there is a possibility that G2AP might suffer an RNA packaging defect that partly contributes to reduced virus infectivity. However, at least two research teams have argued that the gag/pol frameshift signal is not involved in HIV-1 genomic RNA packaging [49,50]. It is unknown whether 2A or ribosomal skipping triggered by 2A disrupts the viral genomic RNA packaging process. Further studies are required to clarify these issues.

Foamy virus (FV) Pol, expressed as a Gag–Pol fusion protein in the absence of spliced Pol, possesses enzymatic activity and the capability to assemble and release as VLPs [51]. However, full-length HIV-1 Gag–Pol expression (i.e., Pol fused to the Gag C-terminus) leads to enhanced Gag–Pol autocleavage, and therefore is incapable of producing VLPs unless PR activity is either suppressed by a HIV-1 protease inhibitor (Figure 3A) or mutationally inactivated [24]. Note that both HIV-1 and FV Gag–Pol possess PR activity, but FV Gag–Pol is capable of forming VLPs while HIV-1 Gag–Pol is not. One possible explanation is that full-length HIV-1 Gag–Pol expresses stronger activity compared to FV Gag–Pol, and therefore is more likely to undergo premature autocleavage prior to VLP assembly. In support of this hypothesis, virus-associated Gag is readily detected in FV [51,52] but barely detectable in HIV-1 [21,53] when equivalent amounts of Gag and Gag–Pol expression vectors are cotransfected.

Even though independent HIV-1 Pol expression from the same gag-coding RNA template can generate infectious virions, it is accompanied by a marked reduction in virus titers. Attenuation of PR activity or reduced Pol expression can significantly increase G2AP virus titers and/or virus production. In this regard, HIV-1 may have evolved a Gag–Pol ribosomal frameshifting mechanism that supports efficient Gag and Pol regulation involving the same RNA template, thereby preventing Pol overexpression by expressing Pol as a Gag–Pol fusion protein at about 5–10% the level for Gag. Gag–Pol fusion protein expression rather than Pol expression also ensures the efficient incorporation of viral enzymes into virus particles. We noted a significant reduction in Pol viral incorporation compared to Gag–Pol, supporting the idea that the N-terminal Gag domain is required for efficient Gag–Pol packaging.

In conclusion, our results suggest that HIV-1 exploits the Gag–Pol ribosomal frameshift mechanism in support of Pol expression as a Gag–Pol fusion protein at relatively low levels, resulting in the promotion of Gag assembly and Pol incorporation into virus particles. HIV-1 virus assembly and replication apparently benefit from this Gag–Pol ribosomal frameshift mechanism.

## Figures and Tables

**Table 1:** Virus titers of HIV-1 mutants.

Construct ^a^	Titer (c.f.u./mL)		Relative Titer	Mean Relative Titer
	Mutant	Dp6*PR ^b^	(%)	(%) ± SD ^c^
D2APol	9	5500	0.164	
	11	5000	0.220	
	8	3750	0.213	
	11	5500	0.200	0.199 ± 0.025
D2A/P22APol	11,000	5500	200.0	
	11,000	5000	220.0	
	9000	3750	240.0	
	8750	5500	159.1	204.8 ± 34.56
D2A/D15EPol	8250	5500	150.0	
	10,500	5000	210.0	
	6250	3750	166.7	
	8250	5500	150.0	169.2 ± 28.33

^a^ Each construct was cotransfected with a VSV-G expression vector into 293T cells. At 48 h post-transfection, supernatants were collected, filtered, and used to infect HeLa cells. Infection and selection of drug-resistant colonies were performed as described in Materials and Methods. ^b^ Dp6*PR titers were determined in parallel experiments performed in quadruplicate. ^c^ Percentage of relative titers were determined by dividing the mutant titer by the Dp6*PR titer in parallel experiments and multiplying by 100%. Mean and standard deviation (SD) values are indicated.

**Table 2:**
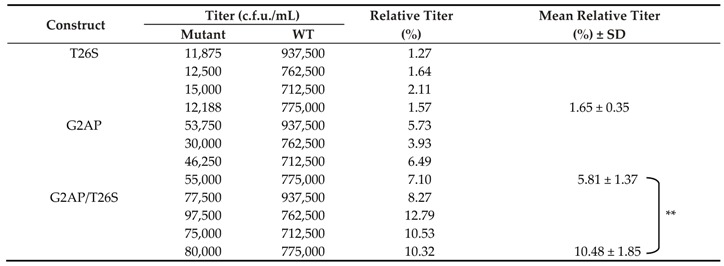
Virus titers of HIV-1 mutants ^a^.

^a^ Wild-type (WT) or each mutant construct was cotransfected with a VSV-G expression vector into 293T cells. Percentage of relative titers were determined as described in Table 1 footnote. Mean and standard deviation (SD) values are indicated. ** *p* < 0.01.

**Table 3:**
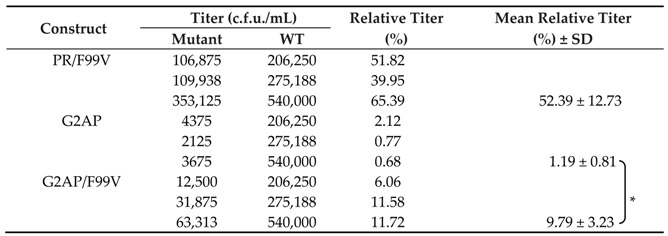
Virus titers of HIV-1 mutants ^a^.

^a^ Virus titers were determined as described in the Table 1 footnote. * *p* < 0.05.
